# Association Between In-Person vs Telehealth Follow-up and Rates of Repeated Hospital Visits Among Patients Seen in the Emergency Department

**DOI:** 10.1001/jamanetworkopen.2022.37783

**Published:** 2022-10-25

**Authors:** Vivek V. Shah, Chad W. Villaflores, Linh H. Chuong, Richard K. Leuchter, Austin S. Kilaru, Sitaram Vangala, Catherine A. Sarkisian

**Affiliations:** 1 Department of Emergency Medicine, Harbor-UCLA Medical Center, Torrance, California; 2 Division of General Internal Medicine and Health Services Research, Department of Medicine, David Geffen School of Medicine at UCLA, Los Angeles, California; 3 Fielding School of Public Health, Department of Health Policy and Management, University of California, Los Angeles; 4 Perelman School of Medicine, Center for Emergency Care Policy and Research, Department of Emergency Medicine, University of Pennsylvania, Philadelphia; 5 Leonard Davis Institute for Health Economics, Wharton School, University of Pennsylvania, Philadelphia; 6 VA Greater Los Angeles Healthcare System, Los Angeles, California

## Abstract

**Importance:**

For patients discharged from the emergency department (ED), timely outpatient in-person follow-up is associated with improved mortality, but the effectiveness of telehealth as follow-up modality is unknown.

**Objective:**

To evaluate whether the rates of ED return visits and hospitalization differ between patients who obtain in-person vs telehealth encounters for post-ED follow-up care.

**Design, Setting, and Participants:**

This retrospective cohort study included adult patients who presented to either of 2 in-system EDs of a single integrated urban academic health system from April 1, 2020, to September 30, 2021; were discharged home; and obtained a follow-up appointment with a primary care physician within 14 days of their index ED visit (15 total days).

**Exposures:**

In-person vs telehealth post–ED discharge follow-up within 14 days.

**Main Outcomes and Measures:**

Multivariable logistic regression was used to estimate the odds of ED return visits (primary outcome) or hospitalization (secondary outcome) within 30 days of an ED visit based on the modality of post–ED discharge follow-up. Models were adjusted for age, sex, primary language, race, ethnicity, Social Vulnerability Index, insurance type, distance to the ED, ambulatory billing codes for the index visit, and the time from ED discharge to follow-up.

**Results:**

Overall, 12 848 patients with 16 987 ED encounters (mean [SD] age, 53 [20] years; 9714 [57%] women; 2009 [12%] Black or African American; 3806 [22%] Hispanic or Latinx; and 9858 [58%] White) were included; 11 818 (70%) obtained in-person follow-up, and 5169 (30%) obtained telehealth follow-up. Overall, 2802 initial ED encounters (17%) led to returns to the ED, and 676 (4%) led to subsequent hospitalization. In adjusted analyses, telehealth vs in-person follow-up visits were associated with increased rates of ED returns (28.3 [95% CI, 11.3-45.3] more ED returns per 1000 encounters) and hospitalizations (10.6 [95% CI, 2.9-18.3] more hospitalizations per 1000 encounters).

**Conclusions and Relevance:**

In this cohort study of patients in an urban integrated health care system, those with telehealth follow-up visits after an ED encounter were more likely to return to the ED and be hospitalized than patients with in-person follow-up. The use of telehealth warrants further evaluation to examine its effectiveness as a modality for continuing care after an initial ED presentation for acute illness.

## Key Points

**Question:**

Do the rates of repeated hospital visits differ between patients who were discharged following an emergency department (ED) visit and have in-person follow-up visits vs those who have telehealth follow-up visits?

**Findings:**

In this cohort study that included 16 987 ED visits, telehealth follow-up was associated with 28.3 more repeated ED encounters and 10.6 more return hospital admissions per 1000 patients compared with in-person follow-up.

**Meaning:**

In this study, telehealth follow-up visits after ED encounters were associated with higher rates of repeated hospital visits even after controlling for presentation acuity, comorbidities, and sociodemographic factors.

## Teaser

This cohort study evaluates whether the rates of emergency department (ED) return visits and hospitalization differ between patients who obtain in-person vs telehealth encounters for post-ED follow-up care.

## Introduction

Nearly 1 in 5 US residents visit the emergency department (ED) annually.^1,2^ An increasing number receive evaluation in the ED and are discharged home without requiring hospitalization.^1,3^ For many patients discharged from the ED, outpatient follow-up is a crucial step that decreases mortality.^3^ Appropriate follow-up may influence decisions on whether patients should be hospitalized. Current efforts to improve care coordination following ED encounters is an active area of policy interest: ensuring patients receive appropriate follow-up while also preventing unnecessary hospitalizations and improving health care value.^1,3,4,5,6,7,8^

Telehealth—the use of synchronous telephone and video technologies and services to provide health care from a distance—underwent rapid adoption since 2020 and now represents 30% of all outpatient care and 34% of all primary care visits.^9,10^ While telehealth may increase availability of follow-up appointments and decrease risk of exposure to communicable diseases, it is possible that telehealth visits may provide suboptimal evaluation in certain scenarios and paradoxically increase the rate that patients return to the hospital.^11,12,13,14^

As a first step toward understanding the effectiveness of post–ED discharge follow-up by telehealth, we examined the association between in-person and telehealth post–ED discharge follow-up visits with subsequent 30-day ED return (primary outcome) and hospitalization (secondary outcome). We hypothesized that the limitations of telemedicine may create challenges for the care of many patients recently discharged from the ED and that telemedicine would be associated with greater subsequent acute hospital utilization compared with patients who obtain in-person follow-up visits.

## Methods

### Study Design

This retrospective cohort study used electronic health record (EHR) data from an urban integrated academic health system in Los Angeles, California, consisting of 2 EDs that provide approximately 150 000 total visits annually. This study followed the Strengthening the Reporting of Observational Studies in Epidemiology (STROBE) reporting guideline and was approved as minimal risk and exempt from the requirement for informed consent by the UCLA institutional review board due to its sole use of deidentified and coded data.

### Study Participants and Patient Characteristics

The cohort included all patients aged 18 years or older who presented to either of the 2 EDs from April 1, 2020, to September 30, 2021, were discharged home thereafter, and completed a follow-up appointment with a primary care physician within 14 days of their index ED visit (15 total days). We selected a 14-day follow-up period given that many acute illnesses would be expected to resolve within that period, with subsequent primary care visits likely related to other issues. We excluded patients who did not have records of a completed follow-up visit within 14 days, returned to the ED before having a follow-up visit, and were not discharged home at the index ED visit (ie, admission, observation, skilled nursing facility, transferred, or expired). We also excluded all patients enrolled in hospice. For patients with multiple ED visits during the study period, each ED encounter was treated as a unique encounter. For example, a patient who was discharged from the ED and had a follow-up visit day 7, then returned day 8 and was again discharged, would count as having only 1 index ED encounter (day 1) and 1 ED return (day 8). If there were a follow-up visit on day 9, they would have 2 index ED encounters (with day 8 serving as both an ED return and a new index ED visit).

To adjust for sociodemographic factors that influence telehealth and ED utilization, we extracted the following patient characteristics from the EHR: age, sex, primary language, self-reported race and ethnicity, Social Vulnerability Index (SVI), insurance type, and distance to the ED.^15,16,17,18^ At this health system, race and ethnicity are self-reported by patients and entered into the EHR at the time of profile creation. Ethnic categories included Hispanic or Latinx or not. Racial categories included American Indian or Alaska Native, Asian, Black or African American, Native Hawaiian or Pacific Islander, White, and other (including unknown and decline to state). To adjust for clinically relevant factors we extracted the Risk Adjustment Factor (RAF) score, a previously tested and widely implemented measure used by the Centers for Medicare & Medicaid Services to calculate risk adjustment, and ambulatory billing (evaluation and management *Current Procedural Terminology*) codes at the index ED visit to adjust for acuity of the initial ED presentation.^19,20^ Ambulatory billing codes were categorized into low acuity (billing levels 1 and 2, including minor or low-to-moderate severity concerns, such as routine wound checks), medium acuity (level 3), and high acuity (levels 4 and 5, including potentially life-threatening conditions, such as active gastrointestinal bleeding).

### Missing Data

**Figure 1.**
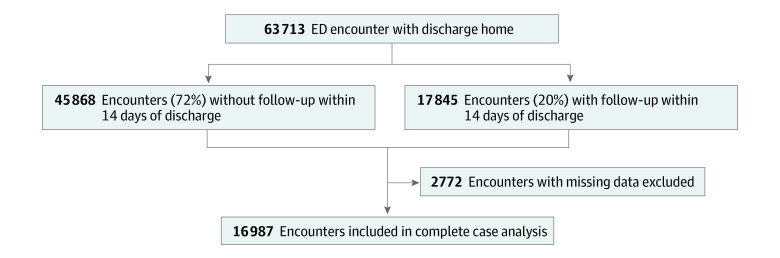
Description of Analytic Sample for Primary Analyses

Missing data ranged from 0% to 14% of the entire sample size, with RAF scores having the highest rate at 14%, followed by SVI at 10%. No values for the independent or dependent variables were missing. We addressed missingness in the SVI by imputing values from median home income and zip code, which are key components of SVI.^18^ RAF scores had the largest odds of missingness for the uninsured population (eTables 1 and 2 in the Supplement). Because of the strong correlation between RAF scores and being uninsured, we conducted 2 sets of analyses: the primary analyses using the entire analytic sample without adjusting for RAF scores and a second sensitivity analysis in which any encounter missing the RAF score was eliminated from the analysis. All other encounters (n = 2772) that were missing data were eliminated from the analytic sample (Figure 1).

### Outcomes

The primary outcome was the rate of ED return visits within 30 days of the follow-up appointment. The secondary outcome was the rate of inpatient hospitalization or observation stays within the same time frame.

### Statistical Analysis

Our primary analyses used multivariable logistic regression to estimate the association between each modality of follow-up with the primary and secondary outcomes. Each model was adjusted for sociodemographic and clinical factors in addition to the time from ED discharge to follow-up. Regressions were clustered by patients to control for patients with multiple ED visits. We conducted secondary analyses on the sample of patients with RAF scores. Results were reported as odds ratios (ORs), average marginal probabilities, and average marginal effects per 1000 patients.^21^

To examine whether secular changes in health care utilization over time could affect our findings, we conducted a sensitivity analysis that used the same regression models from the primary analyses but also included an interaction term between encounter type and time. To assess whether COVID-19 cases were contributing substantially to ED returns or hospitalizations, we also conducted a sensitivity analysis that excluded all patients with COVID-19 at the time of their ED presentation. We calculated E-values^22^ to estimate the minimum strength of association that an unmeasured confounder (such as the pandemic) would need to have with both the treatment and the outcome to fully explain away a specific treatment-outcome association.

To further examine the potential impact of missing RAF scores, we (1) examined sample characteristics within the subgroup of patients with missing RAF scores vs those with RAF scores and (2) measured RAF score missingness by follow-up visit modality across all covariates. To assess whether treating return visits within the 30-day window as independent affected findings, we conducted a sensitivity analysis limiting our analytic sample to 1 index encounter per patient.

A 2-sided *P* ≤ .05 was considered statistically significant. All analyses were performed in Stata version 15.1 (StataCorp).

## Results

Figure 1 illustrates the cohort development from all ED visits between April 1, 2020, and September 30, 2021. There was a total of 12 848 patients with 16 987 ED encounters (mean [SD] age, 53 [20] years; 9714 [57%] women; 2009 [12%] Black or African American; 3806 [22%] Hispanic or Latinx; and 9858 [58%] White): 11 818 (69.6%) in-person follow-up visits and 5169 (30.4%) telehealth follow-up visits. The mean (SD) ages were 54 (21) years for those with in-person follow-up visits and 51 (20) years for those with telehealth follow-up visits; 6557 (55%) of those with in-person follow-up visits were female participants, and 3457 (61%) of those with telehealth follow-up visits were female participants; mean (SD) time-to-follow-up visit was 6 (4) days for both groups (Table 1).

**Table 1.** Study Sample Description

Characteristic	ED encounters by follow-up modality, No. (%)			*P* value
	Total (N = 16 987)	In-person (n = 11 818)	Telehealth (n = 5169)	
Unique patients, No.	12 848	9434	4229	NA
Days to follow-up, mean (SD)	6.2 (4)	6.3 (4)	6.1 (4)	<.001
Patient age, mean (SD), y	53 (20)	54 (21)	51 (20)	<.001
Sex				
Male	7273 (43)	5261 (45)	2012 (39)	<.001
Female	9714 (57)	6557 (55)	3157 (61)	
Ethnicity				
Not Hispanic or Latinx	13 181 (78)	9213 (78)	3968 (77)	.14
Hispanic or Latinx	3806 (22)	2605 (22)	1201 (23)	
Primary language, English				
No	1302 (8)	960 (8)	342 (7)	<.001
Yes	15 685 (92)	10 858 (92)	4827 (93)	
Race				
American Indian or Alaska Native	102 (1)	76 (1)	26 (<1)	.66
Asian	1479 (9)	1008 (9)	471 (9)	
Black or African American	2009 (12)	1422 (12)	587 (11)	
Native Hawaiian or Pacific Islander	46 (<1)	30 (<1)	16 (<1)	
White	9858 (58)	6878 (58)	2980 (58)	
Other^a^	3493 (21)	2404 (20)	1089 (21)	
Primary insurance				
Commercial	9831 (58)	6679 (57)	3152 (61)	<.001
Medicare	5050 (30)	3622 (31)	1428 (28)	
Medicaid	1573 (9)	1120 (9)	453 (9)	
Other insurance	373 (2)	267 (2)	106 (2)	
Uninsured	160 (1)	130 (1)	30 (1)	
Billing level of initial ED encounter^b^				
1	8 (<1)	7 (0)	1 (0)	<.001
2	2622 (16)	1758 (15)	864 (17)	
3	11 116 (65)	7603 (64)	3513 (68)	
4	3098 (18)	2349 (20)	749 (14)	
5	143 (1)	101 (1)	42 (1)	
Distance to emergency department, median (IQR), miles	10 (6-21)	9 (6-20)	11 (7-23)	.002
Social Vulnerability Index, mean (SD)^c^	42 (30)	42 (30)	41 (29)	.28
RAF score, mean (SD)^c^	1 (2)	1 (2)	1 (2)	.40

Abbreviations: ED, emergency department; NA, not applicable; RAF, risk adjustment factor.

^a^ Includes the following responses: other, unknown, and decline to state.

^b^ Billing levels approximate the acuity of the initial ED visit. Greater levels indicate increasing acuity and greater urgency of evaluation.

^c^ Greater values indicate increased social vulnerability or medical complexity (RAF score), respectively.

### Encounter Types Over Time

**Figure 2.**
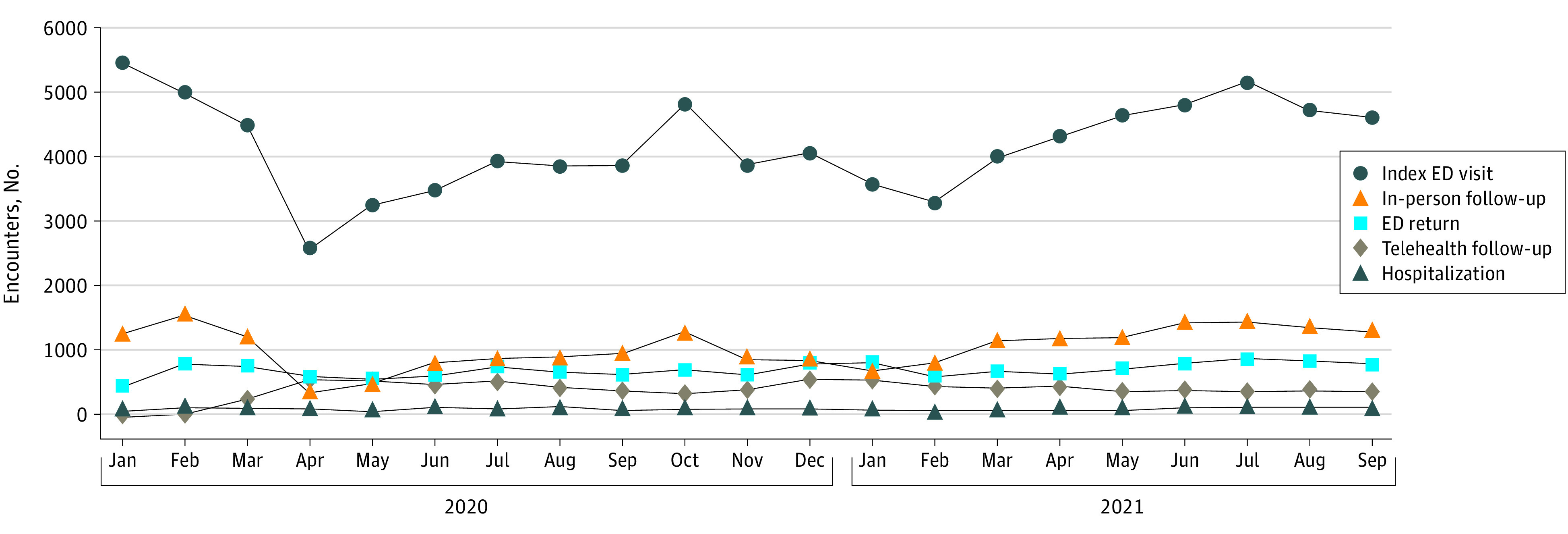
Encounter Types Over Time ED indicates emergency department.

In the analysis that was conducted to examine whether there was a prepandemic trend, we found that post–ED discharge telehealth follow-up visits increased after March 2020 compared with the prepandemic baseline rate (Figure 2). Telehealth visits peaked at 63% (547 of 872) in April 2020 and leveled out to 33% (427 of 1296) by June 2020, remaining stable thereafter. ED return visits increased between April and July 2021, only to decrease again by October 2021. In contrast, hospitalizations remained stable throughout the study period (Figure 2), with a total of 2802 ED returns (17% of encounters) and 676 inpatient admissions (4% of encounters) after follow-up.

### Changes to Rates of ED Returns and Hospitalizations

Among in-person postdischarge follow-up visits, 1865 (16%) were followed by an ED return visit and 438 (4%) with a hospital admission within 30 days. Among telehealth postdischarge follow-up visits, 937 (18%) were followed by an ED return and 238 (5%) with a hospital admission within 30 days.

In the primary analyses, telehealth follow-up was significantly associated with both increased rates of ED returns and hospitalizations. For patients who had a telehealth post–ED discharge follow-up visit compared with those who had an in-person post–ED discharge follow-up visit, the adjusted OR (AOR) for an ED return visit was 1.23 (95% CI, 1.09-1.39) and for hospitalization, 1.31 (95% CI, 1.09-1.58). As average marginal effects, this equates to 28.3 (95% CI, 11.5-45.6) more ED returns per 1000 encounters and 10.6 (95% CI, 2.9-18.4) more hospitalizations per 1000 encounters compared with in-person follow-up visits (Tables 2 and 3).

**Table 2.** ORs, Average Marginal Probabilities, and Average Marginal Effects on ED Returns for 16 987 ED Encounters

ED return	OR (95% CI)	Average marginal		P value
		Probability, %	Effect per 1000 encounters (95% CI)	
Post–ED discharge follow-up visit				
In-person	1 [Reference]	15.6	[Reference]	NA
Telehealth	1.23 (1.09-1.39)	18.5	28.3 (11.3-45.3)	.001
Sex				
Male	1 [Reference]	18.1	[Reference]	NA
Female	0.81 (0.68-0.97)	15.3	−28.5 (−53.0 to −4.0)	.02
Race				
American Indian or Alaska Native	1.63 (0.82-3.24)	24.0	74.8 (−45.2 to 194.8)	.22
Asian	0.75 (0.55-1.03)	13.0	−34.2 (−69.2 to 0.8)	.06
Black or African American	1.22 (0.88-1.70)	19.3	28.4 (−20.0 to 76.8)	.25
Native Hawaiian or Pacific Islander	0.47 (0.16-1.43)	8.7	−78.0 (−165.1 to 9.0)	.08
Other race	0.97 (0.81-1.17)	16.1	−4.0 (−28.4 to 20.4)	.75
White	1 [Reference]	16.5	[Reference]	NA
Ethnicity				
Not Hispanic or Latinx	1 [Reference]	16.6	[Reference]	NA
Hispanic or Latinx	0.98 (0.81-1.1)	16.3	−2.5 (−27.3 to 22.3)	.85
Language, primary				
Not English	1 [Reference]	16.1	[Reference]	NA
English	1.04 (0.83-1.30)	16.5	4.9 (−24.9 to 34.6)	.75
Insurance type				
Commercial insurance	1 [Reference]	12.9	[Reference]	NA
Medicare	1.67 (1.32-2.12)	19.7	68.5 (35.2 to 101.8)	<.001
Medicaid	2.88 (2.23-3.71)	29.5	166.3 (118.5 to 214.2)	<.001
Uninsured	0.77 (0.43-1.39)	10.3	−26.3 (−80.6 to 28.0)	.34
Other insurance	0.89 (0.55-1.41	11.5	−13.6 (−61.8 to 34.7)	.58
First ED visit acuity level				
Low, billing levels 1 and 2	0.99 (0.87-1.14)	17.1	−0.7 (−19.4 to 18.0)	.94
Medium, billing level 3	1 [Reference]	17.1	[Reference]	NA
High, billing levels 4 and 5	0.76 (0.66-0.88)	13.8	−33.8 (−51.2 to −16.5)	<.001
Time period				
April to June 2020	1.04 (0.85-1.27)	16.3	5.4 (−20.6 to 31.3)	.69
July to September 2020	1.03 (0.86-1.24)	16.2	4.0 (−19.3 to 27.4)	.74
October to December 2020	1 [Reference]	15.8	[Reference]	NA
January to March 2021	1.11 (0.93-1.33)	17.2	13.8 (−10.0 to 37.6)	.26
April to June 2021	1.07 (0.90-1.27)	16.6	8.4 (−14.2 to 31.1)	.47
July to September 2021	1.08 (0.90-1.28)	16.8	9.8 (−13.5 to 33.1)	.41
ED				
1	1 [Reference]	15.0	[Reference]	NA
2	1.24 (1.04-1.48)	17.9	28.7 (6.0 to 51.3)	.01
Continuous variables^a^				
Time to follow-up^b^	0.72 (0.59-0.87)	15.6	−4.8 (−7.5 to −2.0)	.001
Social Vulnerability Index	1.00 (1.00-1.00)	16.4	0.6 (0.3 to 1.0)	.001
Patient age, y	1.00 (1.00-1.01	16.5	0.3 (−0.4 to 1.0)	.39
Log distance to hospital	0.98 (0.92-1.05)	16.5	−2.6 (−11.7 to 6.4)	.57

Abbreviations: ED, emergency department; NA, not applicable; OR, odds ratio.

^a^ Average marginal probability calculated at mean of each continuous variable.

^b^ Odds ratio calculated at mean.

**Table 3.** ORs, Average Marginal Probabilities, and Average Marginal Effects on Hospitalizations for 16 987 ED Encounters

Hospitalization	OR (95% CI)	Average marginal		*P* value
		Probability, %	Effect per 1000 encounters (95% CI)	
Post ED discharge follow-up visit				
In-person	1 [Reference]	3.7	[Reference]	NA
Telehealth	1.31 (1.09 to 1.58)	4.7	10.6 (2.9 to 18.3)	.007
Sex				
Male	1 [Reference]	4.8	[Reference]	NA
Female	0.70 (0.57 to 0.85)	3.4	−13.6 (−21.1 to −6.0)	<.001
Race				
American Indian or Alaska Native	1.22 (0.24 to 6.09)	4.6	7.8 (−61.1 to 76.7)	.82
Asian	0.92 (0.66 to 1.29)	3.6	−2.8 (−14.1 to 8.5)	.63
Black or African American	1.16 (0.84 to 1.60)	4.4	5.7 (−7.3 to 18.8)	.39
Native Hawaiian or Pacific Islander	1.75 (0.40 to 7.61)	6.4	26 (−59.1 to 111.0)	.55
Other race	1.10 (0.85 to 1.43)	4.2	3.7 (−6.3 to 13.7)	.47
White	1 [Reference]	3.8	[Reference]	NA
Ethnicity				
Not Hispanic or Latinx	1 [Reference]	3.8	[Reference]	NA
Hispanic or Latinx	1.15 (0.87 to 1.51)	4.4	5.4 (−5.5 to 16.3)	.33
Primary language				
Not English	1 [Reference]	5.3	[Reference]	NA
English	0.70 (0.51 to 0.94)	3.8	−15.3 (−30.0 to −0.8)	.04
Insurance type				
Commercial insurance	1 [Reference]	3.2	[Reference]	NA
Medicare	1.35 (1.04 to 1.74)	4.3	10.5 (1.2 to 19.8)	.03
Medicaid	2.54 (1.82 to 3.55)	7.7	44.4 (23.3 to 65.5)	<.001
Uninsured	1.12 (0.41 to 3.05)	3.6	3.5 (−30.6 to 37.6)	.84
Other insurance	0.46 (0.18 to 1.22)	1.5	−16.9 (−32.2 to −1.8)	.03
First ED visit acuity level				
Low, billing levels 1 and 2	1.60 (1.31 to 1.96)	6.3	22.1 (11.5 to 32.8)	<.001
Medium, billing level 3	1 [Reference]	4.0	[Reference]	NA
High, billing levels 4 and 5	0.44 (0.32 to 0.58)	1.8	−22.2 (−28.4 to −15.9)	<.001
Time period				
April to June 2020	1.20 (0.88 to 1.63)	4.3	6.6 (−5.0 to 18.2)	.26
July to September 2020	1.16 (0.87 to 1.57)	4.2	5.6 (−5.3 to 16.5)	.32
October to December 2020	1 [Reference]	3.6	[Reference]	NA
January to March 2021	0.99 (0.74 to 1.33))	3.6	<0.01 (−10.3 to 10.0)	.96
April to June 2021	1.19 (0,90 to 1.58)	4.3	6.6 (−3.8 to 17.0)	.22
July to September 2021	1.08 (0.81 to 1.43)	3.9	2.6 (−7.5 to 12.7)	.61
ED				
1	1 [Reference]	3.3	[Reference]	NA
2	1.48 (1.21 to 1.80)	4.7	14.5 (7.1 to 22.0)	<.001
Continuous variables^a^				
Time to follow-up^b^	0.69 (0.50 to 0.95)	3.6	−15.1 (−27.5 to −2.7)	.02
Social Vulnerability index	1.00 (1.00 to 1.01)	4.0	0.1 (0.0 to 0.3)	.08
Patient age, y	1.02 (1.01 to 1.03)	3.7	0.7 (0.4 to 0.9)	<.001
Log distance to hospital	1.11 (1.04 to 1.19)	4.0	3.9 (1.4 to 6.5)	.002

Abbreviations: ED, emergency department; NA, not applicable; OR, odds ratio.

^a^ Average marginal probability calculated at mean of each continuous variable.

^b^ Odds ratio calculated at mean.

### Sensitivity Analyses

There was temporal variability with in-person and telehealth follow-up visits that corresponded to surges in local COVID-19 cases (Figure 2). In our sensitivity analysis, a Wald test determined that interacting the modality of follow-up with time period in our study did not add explanatory power (eTable 3 in the Supplement).

In the sensitivity analyses that adjusted for RAF scores in the smaller sample (14 630 encounters, 86% of the larger sample), telehealth follow-up remained associated with greater rates of ED returns, although with a smaller effect size (17.3 [95% CI, 1.1 to 33.5] ED returns per 1000 encounters) (eTable 4 in the Supplement). Telehealth follow-up no longer had a statistically significant association with hospitalizations (6.7 [95% CI, −1.0 to 14.4] hospitalizations per 1000 encounters) (eTable 5 in the Supplement). In both models, RAF scores were associated with large increased rates of ED returns and hospitalizations (41.5 [95% CI, 35.5 to 47.6] ED returns per 1000 encounters; 15.6 [95% CI, 13.6 to 17.6] hospitalizations per 1000 encounters) (eTables 4 and 5 in the Supplement). In the sensitivity analysis limiting our analytic sample to 1 index encounter per patient (n = 12 848), the results were qualitatively unchanged: the observed AOR for ED return in this smaller sample was 1.24 (95% CI, 1.09-1.40), and for hospitalization, it was 1.25 (95% CI, 1.01-1.56).

Restricting *International Statistical Classification of Diseases and Related Health Problems, Tenth Revision *categories to exclude *COVID-19*, *Respiratory signs and symptoms*, or *Other specified upper respiratory infections*, our results were qualitatively unchanged (eTable 6 in the Supplement). The E-value for ED return was 1.76, and for hospitalization, it was 1.95.

Sample characteristics were roughly similar within the RAF missing subgroup compared with the RAF nonmissing subgroup, while ED return and hospitalization were qualitatively lower among the RAF missing group (11% and 2%) compared with the RAF nonmissing group (17% and 4%). RAF scores were missing for 15% of patients with in-person and 10% of patients with telehealth follow-up visits; across all variables, RAF score missing rates were grossly similar between follow-up visit modality (eTable 2 in the Supplement).

### Exploratory Analyses

In exploratory models with separate variables for visit modality, the observed effect size on ED return was qualitatively the same for video visit (AOR, 1.23; 95% CI, 1.07-1.40) and telephone visit (AOR, 1.25; 95% CI, 1.01-1.54). The observed effect size on the outcome of hospitalizations was qualitatively larger for video (AOR, 1.37; 95% CI, 1.13-1.67) than for telephone (AOR, 1.15; 95% CI, 0.80-1.64) (eTable 7 in the Supplement).

## Discussion

In this single integrated health system cohort study using electronic health data, patients who had post–ED discharge telehealth follow-up visits were more likely to return to the ED within 30 days, even after adjusting for sociodemographic factors, acuity of illness, and medical complexity as measured by RAF scores. In addition, patients who had post–ED discharge telehealth follow-up were also more likely to be hospitalized in 30 days compared with patients with in-person follow-up, although after adjusting for RAF scores in the smaller sample this association was not statistically significant. These associations were not moderated by health care utilization fluctuations during the pandemic and were similar after restricting the analysis to non–COVID-19 admissions. While causality cannot be inferred in this observational study, these findings support our hypothesis that the inherent limitations of telemedicine as a modality for caring for patients recently discharged from the ED leads to greater subsequent acute hospital utilization compared with patients who obtain in-person follow-up visits.

These findings need to be considered in the context of a substantial body of science demonstrating the benefits of telemedicine. Earlier work by Jia et al^23^ and Bashshur et al^24^ found that telehealth management of chronic diseases, such as diabetes, chronic obstructive pulmonary disease, and heart failure, can lower the rates of rehospitalization in select patient populations. More recent work has shifted to examining telehealth use in the acute care setting, which differs from chronic disease management in that clinicians are tasked to caring for discrete chief concerns rather than preventative measures around known chronic conditions. These studies have found different findings: a study by Li et al^5^ found that primary care clinics with higher percentage of telehealth usage were associated with increased rates of acute care visits, and a study by Hatef et al^25^ found that primary care telehealth visits for acute and chronic conditions were associated with increased ED encounters overall. Our study builds on this work by looking at post–ED discharge telehealth follow-up across conditions, controlling for patient characteristics as well as secular changes and COVID-19 surges. Unlike the aforementioned studies, in our primary model, we found an increased association between telehealth visits and subsequent hospitalizations, suggesting that the patients with telehealth follow-up who return to the ED might have greater illness severity when they arrive or possibly other medical or social circumstances that prevent ED physicians from being able to discharge them home.

A potential mechanism to explain increased health care utilization after telehealth visits is the inherent limitation in the ability of clinicians to examine patients, which may compel clinicians to have a lower threshold for referring patients back to the ED for an in-person evaluation if they have any ongoing symptoms.^26,27^ It is also possible that independent of the lack of a physical examination, telehealth clinicians may not be able to communicate as well with patients, leading to an inability to fully evaluate or intervene on evolving illness and leading to deterioration in patient condition and subsequent need for hospitalization.

Patients with telehealth visits lived farther from the ED in this study than those with in-person visits. From the patient’s perspective, the remote nature of the encounter may cause them to seek further care for questions or concerns that they were not able to address via telehealth.^28^ Two recent qualitative studies^29,30^ found that physicians believe telehealth is not well suited to evaluate specific concerns, such as chest pain, abdominal pain, or shortness of breath, which represent a large proportion of post-ED follow-up visits. Future qualitative studies might help to determine whether and in what circumstances the return visit modality is being driven by the physicians or the patients (or both).

While telehealth is a relatively new modality of care that allows patients more timely and increased access to care, our study found the even when adjusting for time to follow-up, post–follow-up health care utilization was still higher for patients with telehealth visits. In addition, with the rapid adoption of telehealth, there remain concerns of a growing digital divide.^15,31,32^ While this could skew our results due to inequitable access to and use of telehealth, we attempted to adjust for these effects by controlling for various sociodemographic factors.^15^ As policy makers, health systems, and patients consider how to use telehealth to increase access to care, these findings suggest that telehealth may not be the best modality for all types of encounters, including many post–ED discharge follow-up visits. These findings may have particular relevance for rural health.

Interestingly, the exploratory analyses examining telehealth by modality (video vs telephone) suggested that both video and telephone visits are associated with return to the ED (with similar effect sizes) compared with in-person visits, but for that for the outcome of hospitalizations, video visits have an especially large association compared with in-person visits. These analyses strengthen support for the hypothesis that video visits may be inadequate for acute care follow-up and warrant further investigation.

### Limitations

This study has several limitations. Due to the observational study design, there may be unmeasured factors determining who received in-person vs telehealth visits that could bias our results. For example, discrete EHR data do not contain many of the complex social determinants of health that could have affected our results (eg, unemployment, income, trust). Similarly, while we adjusted for illness acuity, this does not capture how unwell a patient feels or whether the patient has social support and other resources needed for an in-person visit. Second, although we limited the sample to patients who saw a primary care physician, we could not account for patients who followed up outside of this integrated health system, and it is possible that these outside ED visits were unevenly distributed. Third, RAF scores were missing largely in the uninsured population, skewing the sample in the fully adjusted models. Fourth, this study was done in a single academic medical center. Future studies from multiple health systems are needed to determine the generalizability of these findings.

## Conclusions

In this retrospective cohort study, we found that after being discharged from the ED, patients with telehealth post–ED discharge follow-up visits were more likely to return to the ED, even after adjustment for sociodemographic characteristics, insurance type, distance to the ED, severity of illness at the index visit, the time from ED discharge to follow-up, and medical complexity (RAF scores). There were numerically increased subsequent hospitalizations as well, but the difference was not statistically significant. The association of telehealth with increased health care utilization warrants further study to evaluate its appropriateness as modality for post-ED follow-up.

## Notes

Supplement. **eTable 1.** Percentage Missing of Each Covariate **eTable 2.** Odds Ratios and Descriptive Statistics for Participants With Missing RAF Scores **eTable 3.** Wald Test for Interaction Models **eTable 4.** Odds Ratios, Average Marginal Probabilities, and Average Marginal Effects on ED Returns per 1000 Encounters, Adjusting for RAF Scores **eTable 5.** Odds Ratios, Average Marginal Probabilities, and Average Marginal Effects on Hospitalizations per 1000 Encounters, Adjusting for RAF Scores **eTable 6.** Odds Ratios of ED Return Visits and Hospitalizations After Excluding COVID-19 and Related Diagnoses at Index ED Visit **eTable 7.** Adjusted Odds Ratios of ED Returns and Hospitalization by Modality of Telehealth Visit
