# Supplementary material for: Association Between In-Person vs Telehealth Follow-up and Rates of Repeated Hospital Visits Among Patients Seen in the Emergency Department
Source: JAMA Netw Open. 2022 Oct 25;5(10):e2237783. doi: 10.1001/jamanetworkopen.2022.37783 (PMC9597390; doi:10.1001/jamanetworkopen.2022.37783)
Supplement: Supplement. — eTable 1. Percentage Missing of Each Covariate eTable 2. Odds Ratios and Descriptive Statistics for Participants With Missing RAF Scores eTable 3. Wald Test for Interaction Models eTable 4. Odds Ratios, Average Marginal Probabilities, and Average Marginal Effects on ED Returns per 1000 Encounters, Adjusting for RAF Scores eTable 5. Odds Ratios, Average Marginal Probabilities, and Average Marginal Effects on Hospitalizations per 1000 Encounters, Adjusting for RAF Scores eTable 6. Odds Ratios of ED Return Visits and Hospitalizations After Excluding COVID-19 and Related Diagnoses at Index ED Visit eTable 7. Adjusted Odds Ratios of ED Returns and Hospitalization by Modality of Telehealth Visit [file jamanetwopen-e2237783-s001.pdf]

Supplemental Online Content

Shah VV, Villaflores CW, Chuong LH, et al. Association between in-person vs telehealth follow-up and rates of repeated hospital visits among patients seen in the emergency department. *JAMA Netw Open.* 2022;5(10):e2237783. doi:10.1001/jamanetworkopen.2022.37783

eTable 1. Percentage Missing of Each Covariate

eTable 2. Odds Ratios and Descriptive Statistics for Participants With Missing RAF Scores

eTable 3. Wald Test for Interaction Models

eTable 4. Odds Ratios, Average Marginal Probabilities, and Average Marginal Effects on ED Returns per 1000 Encounters, Adjusting for RAF Scores

eTable 5. Odds Ratios, Average Marginal Probabilities, and Average Marginal Effects on Hospitalizations per 1000 Encounters, Adjusting for RAF Scores

eTable 6. Odds Ratios of ED Return Visits and Hospitalizations After Excluding COVID-19 and Related Diagnoses at Index ED Visit

eTable 7. Adjusted Odds Ratios of ED Returns and Hospitalization by Modality of Telehealth Visit

This supplemental material has been provided by the authors to give readers additional information about their work.

© 2022 Shah VV et al. *JAMA Network Open.*

eTable 1. Percentage Missing of Each Covariate

**Variable**	**Missing**	**Total**	**Percent Missing**
Patient Age	0	17,845	0
sex	0	17,845	0
race	306	17,845	1.71
ethnicity	286	17,845	1.6
language	0	17,845	0
Social Vulnerability Index	0	17,845	0
Distance to ED	210	17,845	1.18
First ED Visit Billing Level	90	17,845	0.5
Time Period	0	17,845	0
Specific ED	0	17,845	0
Time to Follow - up	0	17,845	0
RAF Score	2,417	17,845	13.54

© 2022 Shah VV et al. *JAMA Network Open.*

eTable 2. Odds Ratios and Descriptive Statistics for Participants With Missing RAF Scores

eTable 2a: Odd ratio of missing RAF Score

VARIABLES	Missing RAF Score
race = 1, Asian	0.848*
race = 2, Black/African American	0.512***
race = 3, Native Hawaiian/Pacific Islander	0.849
race = 4, American Indian/Alaska Native	0.660
race = 5, Other Race*	0.947
ethnicity = 1, Hispanic/Latinx	0.517***
language = 1, English as Primary Language	0.641***
LogDistance	1.142***
insurance = 2, Medicare	1.068
insurance = 3, Medicaid	2.216***
insurance = 4, Other Insurance	2.529***
insurance = 5, Uninsured	14.12***
PatientAge	0.975***
FirstED_AcuityLevelCode = 2	0.299
FirstED_AcuityLevelCode = 3	0.287
FirstED_AcuityLevelCode = 4	0.422
FirstED_AcuityLevelCode = 5	0.387
Social Vulnerability Index	1.006***
quarter = 3, 07/20-09/20	1.233**
quarter = 4, 10/20-12/20	1.071
quarter = 5, 01/21-03/21	0.842*
quarter = 6, 04/21-06/21	0.956
quarter = 7, 07/21-09/21	1.066
ED = 2	0.660***
Time To Follow Up	1.061***
ED Return	0.573***
Inpatient Admission	0.879
Telehealth	0.640***
Observations	16,987

*includes the following responses: “other,” “unknown,” and “decline to state.”

© 2022 Shah VV et al. *JAMA Network Open.*

Supplement eTable 2b: Descriptive Statistics for Study Population Missing RAF Score			
		Missing RAF Score	NOT Missing RAF Score
Number of Patients		2,058	10,863
Number of Encounters		2,307	14,680
Average Days to Follow-Up (SD)		7 (4)	6 (4)
Average Patient Age (SD)		43 (19)	55 (20)
Encounter Type % (n)			
In-Person Visit		78% (1,790)	68% (10,028)
Telehealth		22% (517)	32% (4,652)
Outcome Measures, % Encounters (n)			
ED return		11% (244)	17% (2558)
Hospitalization		2% (52)	4% (624)
Sex % (n)			
Male		49% (1,135)	42% (6,138)
Female		51% (1,172)	58% (8,542)
Ethnicity % (n)			
Not Hispanic/Latinx		79% (1,820)	77% (11,361)
Hispanic/Latinx		21% (487)	23% (3,319)
Primary Language % (n)			
Not English		8% (178)	8% (1,124)
English		92% (2,129)	92% (13,556)
Race % (n)			
American Indian/Alaska Native		< 1% (9)	< 1% (93)
Asian		9% (219)	9% (1,260)
Black/African American		10% (229)	12% (1,780)
Native Hawaiian/Pacific Islander		< 1% (6)	< 1% (40)
White		58% (1,337)	58% (8,521)
Other Race*		22% (507)	20% (2,986)
Primary Insurance % (n)			
Commercial		55% (1,280)	58% (8,551)
Medicare		17% (396)	32% (4,654)
Medicaid		18% (406)	8% (1,167)
Other Insurance		5% (115)	2% (258)
Uninsured		5% (110)	< 1% (50)

© 2022 Shah VV et al. *JAMA Network Open.*

Billing Level of Initial ED Encounter % (n)			
1		< 1% (5)	< 1% (3)
2		15% (357)	15% (2,265)
3		59% (1,365)	66% (9,751)
4		24% (552)	17% (2,546)
5		1% (28)	< 1% (115)
Median Distance to ED (IQR)		12 (6-32)	10 (6-20)
Social Vulnerability Index (SD)		40 (29)	48 (33)

*includes the following responses: “other,” “unknown,” and “decline to state.”

© 2022 Shah VV et al. *JAMA Network Open.*

Supplement eTable 2c: Descriptive Statistics for Study Population Missing RAF Score		
	Missing RAF Score	
	In-person Visit Cohort (Total n = 11,818)	Telehealth Visit Cohort (Total n = 5,169)
Encounters % of total cohort (n)	15% (1,790)	10% (517)
Outcome Measures % of total encounters (n)		
ED Return	1.6% (185)	1.1% (59)
Hospitalization	3.4% (40)	0.2% (12)
Continuous Variables		
Average Days to Follow-Up (SD)	7 (4)	7 (4)
Average Patient Age (SD)	44 (19)	42 (18)
Median Distance to ED (IQR)	12 (6-29)	14 (6-37)
Mean Social Vulnerability Index (SD)	49 (33)	46 (31)
Sex % of total cohort encounters (n)		
Male	8% (931)	4% (204)
Female	7% (859)	6% (313)
Ethnicity % of total cohort encounters (n)		
Not Hispanic/Latinx	12% (1,429)	8% (391)
Hispanic/Latinx	3% (361)	2% (126)
Priimary Langage % of total encounters (n)		
Not English	1% (134)	1% (44)
English	14% (1,656)	9% (473)
Race % of total cohort encounters (n)		
American Indian/Alaska Native	< 1% (9)	< 1% (0)
Asian	1% (157)	1% (62)
Black/African American	2% (188)	1% (41)
Native Hawaiian/Pacific Islander	< 1% (3)	< 1% (3)
White	9% (1,050)	6% (287)
Other Race*	3% (383)	2% (124)
Primary Insurance % of total cohort encounters (n)		
Commercial	9% (966)	6% (314)

© 2022 Shah VV et al. *JAMA Network Open.*

Medicare	3% (314)	2% (82)
Medicaid	3% (317)	2% (89)
Other Insurance	1% (96)	< 1% (19)
Uninsured	1% (97)	< 1% (13)
Billing Level % of total cohort encounters (n)		
1	< 1% (4)	< 1% (1)
2	2% (250)	2% (107)
3	9% (1,043)	6% (322)
4	4% (471)	2% (81)
5	< 1% (4)	< 1% (6)

© 2022 Shah VV et al. *JAMA Network Open.*

eTable 3. Wald Test for Interaction Models

Outcome	Added parameter	P-Value of Wald Test
ED Recidivism	Encounter Type#Time Period	p = 0.3053
Hospitalization	Encounter Type#Time Period	p = 0.6916

© 2022 Shah VV et al. *JAMA Network Open.*

**eTable 4.** Odds Ratios, Average Marginal Probabilities, and Average Marginal Effects on ED Returns per 1000 Encounters, Adjusting for RAF Scores (N = 14,630)					
ED Return	Odds Ratio	Average marginal probability	Average marginal effect per 1000 encounters	95% Confidence Interval of marginal effect	P-Value of marginal effect
Encounter Type					
In-Person post ED discharge visit	Reference	16.9%	Reference	Reference	Reference
Telehealth post ED discharge visit	1.138	18.6%	17.3	(1.1 - 33.5)	0.037
Sex					
Male	Reference	18.3%	Reference	Reference	Reference
Female	0.891	16.7%	-15.3	(-40.1 – 9.5)	0.227
Race					
American Indian/Alaska Native	1.307	21.5%	38.5	(-45.4 – 122.3)	0.369
Asian	0.733	13.8%	-37.8	(-76.8 – 1.1)	0.057
Black/African American	1.166	19.8%	21.4	(-21.7 – 64.6)	0.330
Native Hawaiian/Pacific Islander	0.548	10.9%	-67.0	(-168.4 – 34.4)	0.195
White	Reference	17.6%	Reference	Reference	Reference
Other*	0.930	16.7%	-9.5	(-35.5 – 17.5)	0.479
Ethnicity					
Not Hispanic/Latinx	Reference	17.6%	Reference	Reference	Reference
Hispanic/Latinx	0.937	16.8%	-8.5	(-34.0 – 16.9)	0.533
Language					
Not English as Primary Language	Reference	16.4%	Reference	Reference	Reference
English as Primary Language	1.093	17.5%	11.4	(-19.4 – 42.3)	0.467
Insurance Type					
Commercial Insurance	Reference	14.7%	Reference	Reference	Reference
Medicare	1.409	19.2%	45.1	(11.9 – 78.3)	0.008
Medicaid	2.445	28.5%	137.6	(86.7 – 188.5)	<0.001
Uninsured					N/A
Other Insurance	0.792	12.2%	-25.5	(-76.4 – 25.5)	0.327
First ED Visit Billing Level					
Low Acuity (Billing Levels 1 and 2	0.830	15.7%	-24.0	(-43.2 – -4.7)	0.015
Medium Acuity (Billing Level 3)	Reference	18.1%	Reference	Reference	Reference
High Acuity (Billing Level 4 and 5)	0.897	16.6%	-14.3	(-33.2 – 4.5)	0.136
Time Period					
04/20-06/20	1.051	17.2%	6.4	(-20.6 – 33.5)	0.641
07/20-09/20	1.048	17.2%	6.0	(-19.0 – 31.0)	0.636
10/20 - 12/20	Reference	16.6%	Reference	Reference	Reference
01/21-03/21	1.146	18.4%	18.0	(-8.1 – 44.1)	0.177
04/21-06/21	1.060	17.3%	7.6	(-16.3 – 31.4)	0.535
07/21-09/21	1.100	17.8%	12.2	(-12.7 – 37.2)	0.338

© 2022 Shah VV et al. *JAMA Network Open.*

Emergency Department					
ED #1	Reference	16.0%	Reference	Reference	Reference
ED #2	1.233	18.7%	27.5	(3.4 – 51.6)	0.025
Continuous Variables**					
Time to Follow-up***	0.687	16.0%	-52.0	(-81.0 - -23.0)	<0.001
Social Vulnerability Index	1.005	17.4%	0.7	(0.3 – 1.1)	<0.001
Patient Age	0.993	17.8%	-0.9	(-1.6 - - 0.2)	0.015
Log Distance to Hospital	0.951	17.4%	-7.0	(-18.3 – 5.0)	0.258
RAF Score	1.370	15.4%	41.5	(35.5 – 47.6)	<0.001

* includes the following responses: “other,” “unknown,” and “decline to state.”

**Average marginal probability calculated at mean of each continuous variable

***Odds ratio calculated at mean

© 2022 Shah VV et al. *JAMA Network Open.*

**eTable 5.** Odds Ratios, Average Marginal Probabilities, and Average Marginal Effects on Hospitalizations per 1000 Encounters, Adjusting for RAF Scores (N = 14,628)					
ED Return	Odds Ratio	Average marginal probability	Average marginal effect per 1000 encounters	95% Confidence Interval of marginal effect	P-Value of marginal effect
Encounter Type					
In-Person post ED discharge visit	Reference	4.0%	Reference	Reference	Reference
Telehealth post ED discharge visit	1.192	4.7%	6.7	(-1.0 – 14.4)	0.086
Sex					
Male	Reference	4.5%	Reference	Reference	Reference
Female	0.875	4.0%	-5.0	(-12.8 – 2.8)	0.209
Race					
White	Reference	4.3%	Reference	Reference	Reference
Asian	0.810	3.6%	-7.3	(-19.5 – 4.9)	0.242
Black/African American	1.022	4.4%	0.8	(-10.0 – 11.7)	0.879
Native Hawaiian/Pacific Islander	2.590	9.5%	52.3	(-64.2 – 168.7)	0.379
American Indian/Alaska Native	0.714	3.2%	-11.1	(-57.6 – 35.4)	0.640
Other*	0.993	4.3%	-0.3	(-11.4 – 10.9)	0.964
Ethnicity					
Not Hispanic/Latinx	Reference	4.2%	Reference	Reference	Reference
Hispanic/Latinx	1.056	4.4%	2.1	(-9.7 – 13.8)	0.732
Language					
Not English as Primary Language	Reference	5.7%	Reference	Reference	Reference
English as Primary Language	0.683	4.1%	-16.0	(-30.8 - -1.3)	0.033
Insurance Type					
Commercial Insurance	Reference	4.0%	Reference	Reference	Reference
Medicare	1.034	4.1%	1.2	(-7.7 – 10.0)	0.739
Medicaid	1.889	6.9%	29.1	(9.0 – 49.2)	0.005
Uninsured	N/A				
Other Insurance	0.214	1.0%	-30.3	(-44.2 - -16.4)	<0.001
First ED Visit Billing Level					
Low Acuity (Billing Levels 1 and 2	1.373	5.6%	13.6	(3.1 – 24.1)	0.011
Medium Acuity (Billing Level 3)	Reference	4.3%	Reference	Reference	Reference
High Acuity (Billing Level 4 and 5)	0.547	2.5%	-17.9	(-25.3 -- -10.5)	<0.001
Time Period					
04/20-06/20	1.189	4.4%	6.2	(-6.2 – 18.7)	0.325
07/20-09/20	1.199	4.5%	6.6	(-5.6 – 18.7)	0.289
10/20 - 12/20	Reference	3.8%	Reference	Reference	Reference
01/21-03/21	1.071	4.0%	2.4	(-8.8 – 13.6)	0.679

© 2022 Shah VV et al. *JAMA Network Open.*

04/21-06/21	1.220	4.5%	7.3	(-3.8 – 18.3)	0.198
07/21-09/21	1.136	4.3%	4.5	(-6.6 – 15.6)	0.425
Emergency Department					
ED #1	Reference	3.5%	Reference	Reference	Reference
ED #2	1.537	5.1%	15.8	(8.0 – 23.7)	<0.001
Continuous Variables**					
Time to Follow-up***	0.699	3.8%	-14.0	(-27.1 -- -0.8)	0.037
Social Vulnerability Index	1.004	4.2%	0.16	(0.0 - 0.3)	0.032
Patient Age	1.006	4.1%	0.22	(0.0 – 0.5)	0.086
Log Distance to Hospital	1.069	4.2%	2.5	(-0.7 – 5.6)	0.126
RAF Score	1.520	2.8%	15.6	(13.6 – 17.6)	<0.001

* includes the following responses: “other,” “unknown,” and “decline to state.”
**Average marginal probability calculated at mean of each continuous variable
***Odds ratio calculated at mean

© 2022 Shah VV et al. *JAMA Network Open.*

eTable 6. Odds Ratios of ED Return Visits and Hospitalizations After Excluding COVID-19 and Related Diagnoses at Index ED Visit

**VARIABLES**	**ED Return**	**Hospitalization**
Telehealth (Reference = In-person Visit)	1.272***	1.356***
Social Vulnerability Index	1.005***	1.003
Patient Age	1.002	1.018***
Female (Reference = Male)	0.815**	0.710***
Race (Reference = White)		
Asian	0.751*	0.950
Black/African American	1.230	1.175
Native Hawaiian/Pacific Islander	0.249**	0.586
American Indian/Alaska Native	1.642	1.000
Other (includes the following responses: "other," "unknown," and "decline to state.")	0.993	1.119
Ethnicity (Reference = not Hispanic/Latinx)		
Hispanic/Latinx	0.981	1.169
English as Primary Language	1.023	0.701**
Insurance (Reference = Commercial Insurance)		
Medicare	1.676***	1.364**
Medicaid	2.912***	2.493***
Uninsured	0.819	1.172
Other Insurance	0.865	0.475
First ED Visit Acuity Level (Reference = Medium Acuity)		
Low Acuity (Billing Levels 1 and 2)	0.956	1.506***
High Acuity (Billing Levels 4 and 5)	0.755***	0.452***
Time Period (Reference = 10/20 - 12/20)		
04/20-06/20	1.032	1.243
07/20-09/20	1.036	1.212
01/21-03/21	1.128	1.047
04/21-06/21	1.063	1.209
07/21-09/21	1.064	1.119
Log Distance to Hospital	0.981	1.125***
ED #2 (Reference = ED #1)	1.232**	1.446***
Time To Follow Up	0.730***	0.710**
Observations	15,979	15,979
*** p<0.01, ** p<0.05, * p<0.1		

© 2022 Shah VV et al. *JAMA Network Open.*

eTable 7. Adjusted Odds Ratios of ED Returns and Hospitalization by Modality of Telehealth Visit

VARIABLES	ED Return	Hospitalization
Video Visit (n = 4,172, Reference = In-person Visit)	1.226***	1.371***
Phone Visit (n = 997, Reference = In-person Visit)	1.251**	1.149
Social Vulnerability Index	1.005***	1.004*
Patient Age	1.002	1.019***
Female (Reference = Male)	0.808**	0.699***
Race (Reference = White)		
American Indian/Alaska Native	1.626	1.229
Asian	0.755*	0.920
Black/African American	1.222	1.165
Native Hawaiian/Pacific Islander	0.471	1.771
Other (includes the following responses: "other," "unknown," and "decline to state.")	0.970	1.103
Ethnicity (Reference = not Hispanic/Latinx)		
Hispanic/Latinx	0.982	1.151
English as Primary Language	1.038	0.694**
Insurance (Reference = Commercial Insurance)		
Medicare	1.674***	1.349**
Medicaid	2.879***	2.562***
Uninsured	0.880	0.463
Other Insurance	0.770	1.120
First ED Visit Acuity Level (Reference = Medium Acuity)		
Low Acuity (Billing Levels 1 and 2)	0.995	1.601***
High Acuity (Billing Levels 4 and 5)	0.764***	0.436***
Time Period (Reference = 10/20 - 12/20)		
04/20-06/20	1.042	1.201
07/20-09/20	1.031	1.167
01/21-03/21	1.110	0.988
04/21-06/21	1.067	1.191
07/21-09/21	1.078	1.072
Log Distance to Hospital	0.981	1.110***
ED #2 (Reference = ED #1)	1.242**	1.483***
Time To Follow Up	0.716***	0.685**
Observations	16,987	16,987
*** p<0.01, ** p<0.05, * p<0.1		

© 2022 Shah VV et al. *JAMA Network Open.*
